# Pulmonary Artery Aneurysm: A Rarity and Surgical Enigma

**DOI:** 10.7759/cureus.38157

**Published:** 2023-04-26

**Authors:** Joel Badders, Patrick Roughneen, Navyatha Mohan, Erin Roughneen

**Affiliations:** 1 School of Medicine, University of Texas Medical Branch, Galveston, USA; 2 Cardiothoracic Surgery, University of Texas Medical Branch, Galveston, USA; 3 Surgery, University of Texas Medical Branch, Galveston, USA; 4 Biology, Southern Methodist University, Dallas, USA

**Keywords:** thoracic aneurysm, aneurysmectomy, atypical chest pain, thoracic surgery, surgical indications, pulmonary artery aneurysm

## Abstract

Given the low incidence of pulmonary artery aneurysms (PAAs), proven surgical indications do not yet exist. We present a patient with a 6.3 cm PAA who underwent an open sternotomy, pulmonary artery aneurysmectomy, and repair with an aortic homograft. We discuss surgical indications, including pain, growth in diameter, and diameter of 5.5 cm and larger. The current recommended surgical indication for the size of PAAs is based on recommendations for aortic aneurysms and observation in a small number of operable patients, highlighting the need for more discussion and reporting of this rare presentation.

## Introduction

A clinician would be surprised to discover a pulmonary artery aneurysm (PAA), given their historically uncommon presentation. A 1947 autopsy review uncovered only eight cases of PAAs in 109,571 examinations [1]. Given the low incidence of PAA presentation, as well as the even rare instances of surgical repair [2], many institutions lack enough exposure to gain knowledge and experience in comfortably treating them. Indications for surgical repair may be enigmatic initially. Much like aortic aneurysms, PAAs can go undetected and yet pose a risk of rupture or dissection [2], highlighting the need for reports of the condition, surgical repairs, and establishment of treatment guidelines. This case provides a detailed report of a successful fourth redo sternotomy of a large PAA to add to the small but growing number of reports on the matter.

## Case presentation

The patient was found to have critical pulmonary stenosis at 18 months of age after presenting with cyanosis and dyspnea. She underwent a sternotomy and Brock procedure, a technique involving a blind incision of the pulmonary valve, which successfully relieved the symptoms. Her symptoms recurred at the age of six. Thus, she underwent a second sternotomy, open pulmonary valvuloplasty, and repair of an incidentally found atrial septal defect, after which the patient could tolerate mild exercise for the duration of her childhood. At the age of 18, the patient experienced sudden bilateral vision loss, slurred speech, and gait difficulties. She was diagnosed with a paradoxical embolus secondary to right to left shunting with an open atrial septal defect. She subsequently underwent a sternotomy and atrial septal defect repair with a Dacron patch.

For three years, the patient was being monitored at our institution every six months for an ascending aortic aneurysm measuring 4.4 cm, a main PAA measuring 5.2 cm, and atrial fibrillation. In addition, during this period, she underwent frequent echocardiograms, blood pressure management with various beta blockers, and yearly cardiac MRIs. The size of the aortic aneurysm and PAA remained unchanged during this period.

**Figure 1:**
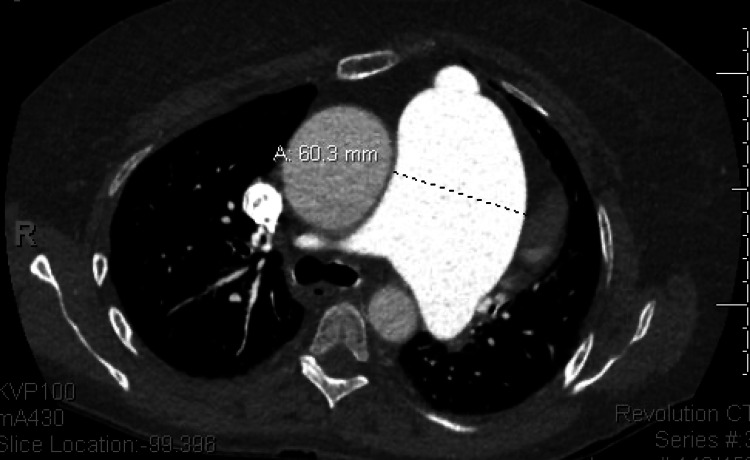
CT thorax with contrast showing a PAA measuring 60.3 mm (6.3 cm) PAA: pulmonary artery aneurysm

At age 61, the patient presented to the emergency department with sharp, sub-sternal chest pain. Two different troponin levels were within normal limits. An EKG showed no changes compared to an EKG four months prior, which was conducted to monitor atrial fibrillation. Further workup included a “triple rule out” thoracic CT scan, which revealed patent coronary arteries, a PAA measuring 6.3 cm with no dilation of the right and left branches (Figure 1), with a 1.1 cm interval expansion in size compared to a cardiac MRI conducted two years prior, as well as the previously mentioned ascending aortic aneurysm which was unchanged in size.

**Figure 2:**
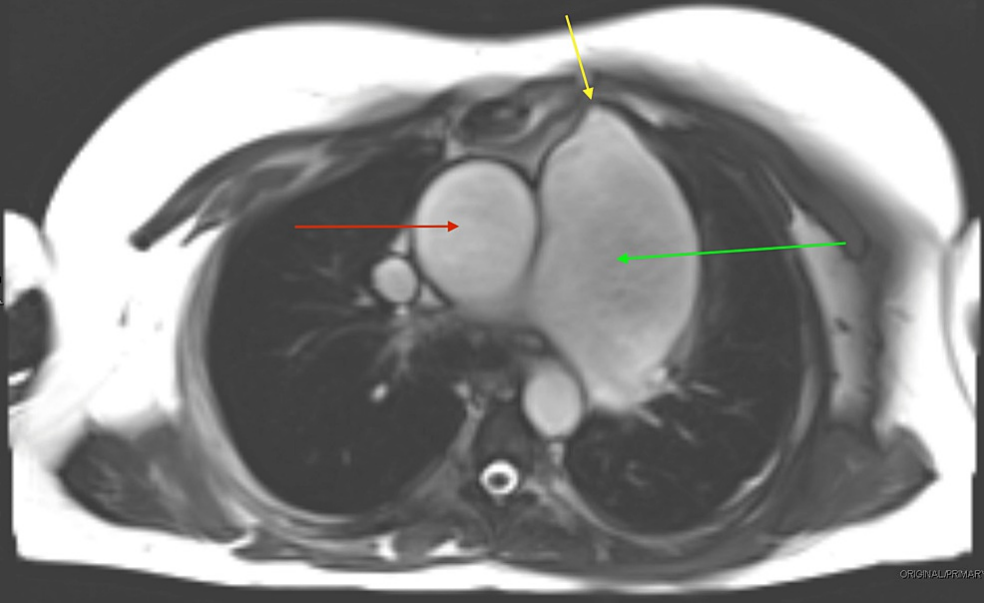
Morphological cardiac MRI (1.5 T with gadolinium contrast) showing erosion of the PAA into the sternum Yellow arrow: pulmonary artery eroding into the sternum Green arrow: PAA as previously appreciated Red arrow: aortic aneurysm

A transthoracic echocardiogram showed an ejection fraction of 45-50%, a severely dilated right atrium, a right ventricular systolic pressure of 35-40 mmHg, and a Doppler aliasing across the pulmonic valve. No other structural or physiologic changes were noted compared to a previous echocardiogram. The pulmonic valve Doppler aliasing was believed to suggest pulmonic valve stenosis or right ventricular outflow obstruction. Given the apparent growth in the size of the PAA (6.3 cm compared to 5.2 cm on imaging two years prior), surgical evaluation and more extensive cardiac workup were recommended. A cardiac MRI showed erosion of the PAA into the sternum (Figure 2). A pharmacologic stress test showed no abnormalities. Left heart catheterization and coronary angiography showed no abnormalities. Right heart catheterization showed increased right heart filling pressures, a mean pulmonary artery pressure of 27 mmHg, and a pulmonary vascular resistance of 4.5 Woods Units, findings consistent with pulmonary hypertension.

After extensive risk assessment, surgical repair of the PAA was recommended and discussed, and the patient decided to undergo the operation. At surgery, the existing sternal wires were removed, and the sternum was divided in a standard fashion. The femoral vessels were cannulated, the heart was dissected, and a cardiopulmonary bypass was initiated. The aorta was cross clamped, and the right ventricle and the PAA were opened. We inspected the right ventricular outflow tract (RVOT) for muscle bands and dissected infundibular hypertrophied muscle bands. A 23 mm aortic homograft was utilized and sewn into position in a running fashion, with the dissected pulmonary artery encompassing the graft. The aortic leaflet of the homograft was used to fashion a patch on the RVOT and was thus utilized to reconstruct where muscle band dissection occurred. The patient was weaned off bypass successfully, but perioperative bleeding necessitated using a temporary Esmarch bandage for sternal closure. Permanent closure with sternal wires was delayed until the next day and was uncomplicated.

**Figure 3:**
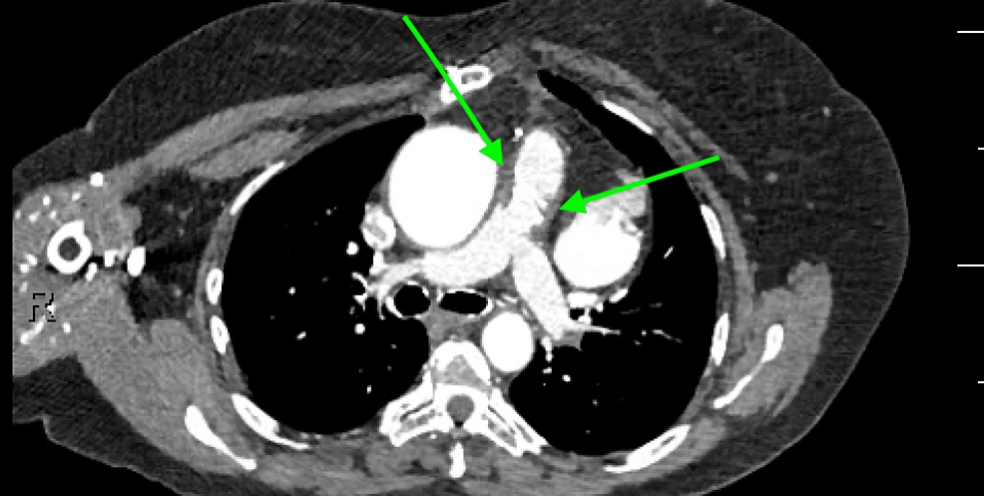
CT thorax with contrast. One-year postoperative evaluation showing stable pulmonary artery diameter and post-reconstruction changes compared to immediate postoperative evaluation Green arrows: post-reconstruction changes

The patient was stable postoperatively, and she was discharged on postoperative day eight. She gradually improved to her pre-surgical functional status on outpatient follow-up and was noted to have a resolution of chest pain. She continues to do well three years later and has maintained New York Heart Association Class I status (Figure 3).

## Discussion

Current literature categorizes PAAs on their etiology. PAAs secondary to congenital heart disease comprise the majority of reported cases, with patent ductus arteriosus, ventricular septal defect, and atrial septal defect being the most frequently described causes [3]. PAAs may also occur in the setting of pulmonary hypertension [4], which likely plays a role in the dilation of the pulmonary artery. In addition, PAAs due to infectious causes, such as syphilis, tuberculosis, and mucormycosis, have been described in reports [5]. Lastly, PAAs can be defined as idiopathic in the absence of any clear etiology [6]. The case presented here occurred in the setting of pulmonary hypertension, likely due to multifactorial causes of congenital heart disease and extensive cardiac history.

PAAs treated surgically constitute a small number of the cases discovered. A retrospective study by Hou et al. discovered 21 PAA cases over a 35-year period, of which only five were selected for surgical intervention [2]. Choosing surgical intervention versus more conservative management requires a thorough assessment of patient history and presentation. Indications for conservative management include stable size and asymptomatic presentation. These patients should undergo close cardiac follow-up and yearly serial imaging, with shorter time intervals for imaging if clinically indicated. In the absence of other potential causes, pain should be a strong indication for PAA surgical repair, as it may result from pulmonary artery expansion and triggering of nociceptors in the vessel wall and suggest a risk of rupture [2]. The chest pain of our patient was theorized to be generated by PAA erosion into the sternum and strongly indicated surgical repair. Pulmonary artery hypertension should also be investigated thoroughly and should encourage the clinician to consider surgery [7].

Strong evidence for surgical indications of PAAs based purely on size does not yet exist. Low incidence makes an association between a specific size and poor outcomes difficult to establish. Kreibich et al. recommend surgical repair for PAAs of 5.5 cm in line with current recommendations for aortic aneurysms [7], a much more common condition in which a size threshold associated with poor outcomes is clearly understood. However, the pulmonary artery constitutes a lower-pressure system and has thinner walls compared to the aorta [8]. Thinner walls make a significant difference in the ability of the pulmonary artery to withstand escalation of wall stress resulting from increased intra-lumenal pressure and vascular dilatation compared to the aorta. The law of Laplace can be simplified and extrapolated to the physiology of aneurysms in blood vessels through the following equation:

Wall Tension (T) = Transmural Pressure (P) x Radius (R)/wall thickness (w)

The inverse relationship between wall thickness and wall stress explains the following concept: widening in diameter of a blood vessel and consequential narrowing of the vessel wall leads to higher wall tensions and subsequent increase in further stretching [9]. Moreover, Mower et al. explain that while the law of Laplace describes this relationship, it cannot be used to provide detailed information on stress distribution with an aneurysm wall [10]. In their performance of finite element analysis on aortic aneurysm models, they discovered that decreasing wall thickness by one-half increased maximal wall stress by 390% along the circumferential axis [10]. The authors assert that wall thickness may be more critical than aneurysm diameter in producing changes in wall stress. The importance of wall thickness in aneurysm rupture may be underestimated by an oversimplified approximation from the law of Laplace [10]. These findings show that the thinner walls of the pulmonary artery diminish its potential for radial expansion in comparison to the aorta. While size indications for PAAs based on indications for aortic aneurysms serve as an improvised solution in the absence of sufficient reporting of PAAs, the difference in wall thickness between the two vessels emphasizes the need to establish a threshold size for the surgical indication of PAAs based on reporting of the condition and associated outcomes.

Deb et al. take a different approach by asserting that cystic medial degeneration (CMD) is the most crucial factor in the pathogenesis of idiopathic PAAs [11]. Furthermore, they recommend repairing PAAs greater than 6.0 cm because of an observed association between CMD and PAAs of such size [11]. While CMD may be an essential factor in the pathogenesis of PAAs, we assert that hemodynamic stress on the vessel walls may reach dangerous levels even in the absence of CMD.

Aneurysmorrhaphy provides one option for repairing PAAs, which includes excision of the dilated vessel wall and reconstruction by overlapping the native vessel walls and re-suturing the defect shut. Another option is aneurysmectomy and replacement of the removed vessel with an allograft, synthetic graft, or xenograft [2,8,11]. A comprehensive comparison of long-term outcomes between the two main options does not yet exist, and usage is primarily based on surgeon preference. However, aneurysmorrhaphy only decreases the diameter of the vessel and does not address existing abnormalities of the vessel wall [8]. Therefore, we recommend aneurysmectomy and replacement of the PAA with a homograft, as the native vessel may have already experienced CMD or significant wall thinning and stress.

## Conclusions

Aneurysms of the pulmonary artery continue to be an enigmatic presentation. Based on the case of our patient and a review of previously reported cases, we recommend surgical repair for PAAs associated with pain, growth in the size of 0.5 cm in six months, in the setting of pulmonary hypertension, and PAAs measuring 5.5 cm in diameter or greater as recommended by other publications. Limited reporting makes clinically proven surgical indications based on size alone challenging to establish. We encourage reporting of surgical and conservative treatments of PAAs and their outcomes to confirm clinically relevant guidelines and size indications for surgical management.
